# Islet inflammation in type 2 diabetes

**DOI:** 10.1007/s00281-019-00745-4

**Published:** 2019-04-15

**Authors:** Marianne Böni-Schnetzler, Daniel T. Meier

**Affiliations:** 1 Endocrinology, Diabetes and Metabolism, University Hospital of Basel, 4031 Basel, Switzerland; 2 Department of Biomedicine, University Hospital and University of Basel, Hebelstrasse 20, 4031 Basel, Switzerland

**Keywords:** Islet inflammation, Type 2 diabetes, IL-1β, Insulin, Cytokines, β-Cell

## Abstract

Metabolic diseases including type 2 diabetes are associated with meta-inflammation. β-Cell failure is a major component of the pathogenesis of type 2 diabetes. It is now well established that increased numbers of innate immune cells, cytokines, and chemokines have detrimental effects on islets in these chronic conditions. Recently, evidence emerged which points to initially adaptive and restorative functions of inflammatory factors and immune cells in metabolism. In the following review, we provide an overview on the features of islet inflammation in diabetes and models of prediabetes. We separately emphasize what is known on islet inflammation in humans and focus on in vivo animal models and how they are used to elucidate mechanistic aspects of islet inflammation. Further, we discuss the recently emerging physiologic signaling role of cytokines during adaptation and normal function of islet cells.

## Introduction

Type 2 diabetes (T2D) is a chronic progressive disease associated with obesity and insulin resistance. The onset of T2D is mainly determined by the progressive failure of the pancreatic islet β-cells to secrete sufficient levels of insulin to maintain normoglycemia [1, 2]. Numerous preclinical and clinical studies showed a causal link between sterile low-grade inflammation and metabolic diseases including T2D [3–5]. Acute inflammation in response to pathogens and irritants typically starts at the site of an insult with extravasation of plasma and leukocytes followed by a cellular phase dominated by granulocytes. In contrast, chronic inflammation in metabolic diseases and obesity lacks an acute immunovascular phase and mainly involves mononuclear cells. There is typically a 2- to 3-fold increase of proinflammatory cyto- and chemokines that are not confined to a particular site but manifests in whole organ systems, such as liver, fat, kidney, eye, heart, and pancreatic islets. In islets, activation of the innate immune system contributes to the reduction of β-cell mass and function [6, 7]. This activation is characterized by elevated innate immune cells and proinflammatory mediators. In the following, we will separately review the current knowledge on islet inflammation in humans with T2D and in vivo rodent models subjected to various anti-inflammatory treatments.

## Immune cell infiltration in T2D islets

### Immune cell infiltration in T2D in human islets

Low-grade islet inflammation is considered to be part of the aetiopathology of T2D. Supporting this concept, elevated numbers of immune cells are observed in islets of humans with T2D (Table 1). While insulitis was an established hallmark of type 1 diabetes (T1D) for many decades, it was first described in T2D only in 2007 [9]. Ehses et al. observed increased numbers of macrophage marker CD68+ cells in and around islets when comparing histological sections from 7 non-diabetic and 8 T2D individuals. These islet-associated CD68+ cells were also positive for the resident tissue macrophage marker CD163 and for HLA-2 and were not located in the vicinity of apoptotic β-cells. Further, there was no increase in panT-cell marker-CD3+ cells or granulocytes. This seminal finding of increased macrophage numbers in T2D islets was confirmed in a second study with a larger sample size, consisting of pancreas sections from 15 T2D and 16 non-diabetic cases stained for CD68 [11]. In this study, 20.6% of the islets derived from patients with diabetes contained more than 3 CD68+ cells/islet while in non-diabetic cases it was 4.6%.

**Table 1:** Cytokines and immune cells in islets of patients with T2D

Islet source	Sample size	Method	Immune cells	Cytokines	Ref.
Pancreas sections	5 T2D	Immuno-histochemistry In situ hybridization	n.a.	IL-1β↑ (22.5% of islets from T2D were positive)	[8]
Pancreas sections	9 T2D 7 controls	Immuno-histochemistry	CD68+↑ CD3+ T- cells→ Granulocytes→	n.a.	[9]
β-cell enriched samples isolated by LCM of pancreas sections	10 T2D 9 controls	Gene array qPCR	n.a.	IL-1β: gene array↑ qPCR↑ IL-8: gene array↑ qPCR↑ (n.s.)	[10]
Pancreas section of T2D patients	15 T2D 16 controls	Immuno-histochemistry	CD68+↑	n.a.	[11]
β-cell enriched samples isolated by LCM of pancreas sections	10 T2D 10 controls	Gene array Immuno-histochemistry of CCL2	n.a.	CCL13, CCL2↑ IL-1β, IL-8, CCL11, CXCL1, IL6↑ (n.s.)	[12]
Isolated islets	10 T2D 38 controls	Gene array, co-expression networks	n.a.	IL6, IL11, IL33, IL13RA2, IL18R1, IL1R1, IL1R2, IL1RL1↑	[13]
Pancreas sections	20 T2D no amyloid 26 with amyloid 20 controls	Immuno-histochemistry	CD68+↑ selectively in amyloid + samples and not in amyloid − samples CD163+→	n.a.	[14]
Isolated islets cultured for ≅ 3 days	18 T2D 21 controls	FACS islet function	CD45+↑ CD3+ T-cells→ CD20+ B-cells↑	CCL2, TNF-α↑	[15]
Pancreas sections	11 T2D 15 controls	Immuno-histochemistry	CD8+ T-cells↑ (only in exocrine tissue)	n.a.	[16]
Pancreas sections	7 T2D 7 controls	Electron-microscopy	Macrophages↑ Lymphocytes→ Mast cells→	n.a.	[17]
Isolated islets	4 T2D 6 controls	Single cell RNAseq	n.a.	Cytokine signaling in acinar cells↑	[18]
Pancreas sections	17 T2D 16 controls	Immuno-histochemistry	CD45+↑	n.a.	[19]
Pancreas sections	50 T2D 44 controls	Immuno-histochemistry	28% T2D with insulitis CD45+ (endo- and exocrine)↑	n.a.	[20]
Isolated islets SR and LCM of β-cell enriched samples	Islets: 19 T2D 84 controls SR: 36 T2D 32 controls	Gene array	n.a.	Islets: IL-1β, CCL26, CCL3, CCL8, CXCL1, CXCL11, CXCL12, CXL2, CXCR7↑ SR→	[21]

*Ref.* reference; *LCM* laser capture microdissection; *n.a.* not analyzed; *SR* surgical resection

↑ significantly increased; → unchanged; ↑ (n.s) trend to increase, not significant

In several later histological investigations and in one study using FACS analysis of dispersed islet cells, the numbers and subtypes of immune cells infiltrating T2D islets were further characterized [14–17, 19]. Kamata et al. analyzed 46 T2D and 20 non-diabetic cases. Of the 46 cases with T2D, 26 showed amyloid deposits in their islets and interestingly, only islets from amyloid-positive cases presented with increased macrophage marker CD68+ cells while islets from non-diabetic and T2D cases lacking amyloid had normal CD68+ cell numbers. In the amyloid-positive cases, CD68 and iNOS double-positive cells (likely proinflammatory M1 polarized macrophages) predominated over CD163 and CD204 double-positive cells (likely tissue repair-oriented M2 polarized macrophages), pointing to proinflammatory macrophage activation in human T2D islets [14]. Rodriguez-Calvo et al. analyzed 11 T2D and 15 non-diabetic pancreas sections stained for T cell markers CD8 and CD4 and myeloid lineage marker CD11c [16]. They observed a higher CD8 infiltration in the exocrine tissue and the peri-islet area in T2D pancreata, but not within islets, suggesting that the exocrine gland is also infiltrated with immune cells in T2D. Using isolated and dispersed islets and FACS analysis, Butcher et al. found increased total numbers of resident leucocytes (pan immune cell marker CD45+ cells) including CD11b+CD11c+ myeloid cells in T2D islets. Interestingly, CD20+ B-cell numbers were increased as well, although they were low in total number. Islet T-cell numbers (CD3+) were not changed in T2D islets confirming previous reports. A comparison of the numbers of CD45+ cells in T2D islets with preserved insulin secretion (5 cases) to those which are completely dysfunctional (5 cases) revealed that only islets with preserved function displayed increased CD45 numbers [15]. This could hint a temporal increase of immune cells prior to the demise of β-cell function. Elevated numbers of CD45-positive cells within islets and with peri-islet localization were also observed in sections from 17 T2D and 16 non-diabetic cases [19]. A recent publication by Lundberg et al. compared the extent of islet inflammation in histological sections of 50 T2D, 13 T1D, and 44 healthy controls, also using the CD45 pan-immune cell marker [20]. Remarkably, the extent of insulitis [using consensus definition of insulitis for T1D [22]] was very similar between T2D with 28% and T1D with 31% of the cases. However, a major difference in insulitis between T1D and T2D was that in T2D, the CD45+ immune cells were mainly macrophages whereas in T1D, they were mainly T-cells [17, 20].

Taken together, an accumulating number of studies using histological sections and isolated islet from humans show that insulitis characterized by increased macrophage infiltration in the islet is a feature of islet pathology in human T2D.

### Immune cell infiltration in rodent models of T2D

As observed in human pancreas sections of T2D, numerous studies with rodent models of T2D show increased macrophage infiltration in islets [9, 23–28]. While human histology studies remain observational, the use of rodent models allows for elucidation of the underlying mechanisms causing islet immune cell infiltration. Further, the types and sources of infiltrating immune cells can be investigated in more detail. Increased numbers of both macrophages and granulocytes were described for the first time in the GK rat, a spontaneous, non-obese model of T2D [9, 24]. Infiltration of CD68+, MHCII+, and CD53+ immune cells into islets of GK rats was prevented by treatment with the IL-1Receptor antagonist (IL-1Ra) [27]. This also improved glycemia and insulin secretion, implicating the activation of the IL-1 pathway in islet immune cell infiltration and β-cell dysfunction. Similar observations were made in a mouse model with islet inflammation induced by high-fat-diet feeding in combination with activation of the renin-angiotensin system [26]. Treatment with a specific anti-IL-1β antibody diminished islet infiltration with CD45+ immune cells and led to improved insulin secretion and blood glucose control [26]. Egushi et al. used the severely obese db/db mouse model, the high-fat-diet-fed KKAy mouse, and mice infused with the saturated fatty acid palmitate to demonstrate increased islet infiltration with CD11b+Ly-6C+ macrophages, which have a proinflammatory M1 phenotype [23]. Further, by depleting macrophages with clodronate-containing liposomes, which ameliorated β-cell dysfunction, they provide evidence for causal role of these proinflammatory-skewed islet macrophages for β-cell dysfunction [23]. These findings are supported by another study with the Zucker diabetic fatty rat where islet inflammation and β-cell demise were promoted by endocannabinoids. Macrophage-specific deletion of the endocannabinoid receptor CB_1_R or depletion of macrophages protected from islet inflammation and β-cell failure [25]. It is still unclear if these macrophages are recruited to the islets [23] or result from proliferation of islet resident macrophages [29].

Taken together, different rodent models of T2D display an increased number of infiltrating islet macrophages with a polarity shift towards a proinflammatory state which is causally linked to β-cell dysfunction and loss of β-cell mass.

## Cytokines in T2D islets

### Cytokines in human T2D islets

Hormones, the main islet product, act both locally and in distant locations and can be sampled in the blood. In contrast, cytokines and chemokines usually act in a paracrine manner, and therefore, human islet cytokines can only be studied either in isolated and cultured human islets from cadaver donors or in pancreas sections from organ donors or pancreatectomized patients. However, material for such investigations is scarce and there is great variability in the acquisition of the samples. Traditionally, a cocktail of proinflammatory factors consisting of IL-1β, TNF-α, and interferon-γ has been used in islet cultures in the context of autoimmune destruction of β-cells in T1D [30, 31]. In 2002, IL-1β, a master regulator and amplifier of immunological responses, was first observed by immunohistochemistry and in situ hybridization in histological sections from 5 patients with poorly controlled T2D [8]. Further, in human islet cultures, β-cell dysfunction induced by glucotoxicity was partially reversed by antagonizing this islet-derived IL-1β with anakinra, a recombinant human IL-1Ra. Interestingly, endogenous IL-1Ra expression was decreased in β-cells in histological sections from T2D patients [32]. Exposure of human islets to high glucose reduced IL-1Ra and increased IL-1β expression which shifts the ratio of IL-1β to IL-1Ra in favor of the proinflammatory IL-1β [10, 33]. This points to an imbalanced and thus activated IL-1 system in human islets in T2D. Furthermore, treatment of human islet cultures with IL-1Ra almost completely prevented the induction of proinflammatory factors IL-6, IL-8, IL-1β, CXCL1, CCL2, and TNF-α induced by a diabetic milieu (fatty acid and/or glucose) or by activation of toll-like receptors (TLR) 2 and 4 [12, 34]. This indicates that these cytokines are downstream of IL-1Receptor activation and that IL-1β governs cyto- and chemokine expression in human islets. Further, a later study with cultured islets from T2D subjects revealed a negative correlation between β-cell function and expression of *TNF* and *CCL2*, further implicating elevated cyto- and chemokines in islet dysfunction in T2D [15].

Transcriptome studies provided conflicting results concerning the differential expression of proinflammatory factors in islet specimens from T2D patients. An initial microarray study revealed no difference [35] while later studies report an increase in the expression of various cytokines in T2D islet [13, 15, 36]. Of note, Mahdi et al. identified in islets from T2D patients a group of coexpressed genes, which was enriched for IL-1-related genes and which was associated with impaired insulin secretion. These genes included not only the cytokines *IL11*, *IL33*, *IL24*, and *IL6* but also the IL-1 family receptors *IL18R1*, *IL1R1*, the IL33 receptor (*IL1RL1*), and the decoy receptor *IL1R2* [13, 37]. Possible reasons for the variable results obtained from isolated islets are differences in the enzymatic digestion procedures of the pancreatic tissue, which is known to induce inflammation [38], disparities in the procedure and duration of the recovery and culture of the islets, and technical discrepancies in the assays used to assess gene expression. Analysis of specimens harvested from frozen tissue sections by laser capture micro-dissection may overcome these limitations. Indeed, the genes encoding IL-1β and IL-8 were found to be upregulated in a gene array screening and qPCR validation when comparing β-cell-enriched samples from 10 T2D patients to 9 from control organ donors [10]. In addition, the same gene arrays revealed increased *CCL2* and *CCL13* in T2D islets [12]. On the other hand, a recent transcriptome study using specimens from surgical pancreas resections did not confirm presence of elevated proinflammatory factors in β-cell-enriched fractions [21]. However, the same study also assessed whole islets (which still contain immune cells) and confirmed the presence of increased gene expression of proinflammatory factors IL-1β, CCL26, CCL3, CCL8, CXCL1, CXCL2, CXCL11, and CXCL12. Noteworthy, the fraction of immune cells contained in isolated whole islets and in β-cell-enriched samples is unknown. Different proportions of immune cells in the specimens may contribute to variable cyto- and chemokine expression in unequally isolated samples. Indeed, islet immune cell numbers are increased and skewed to a proinflammatory phenotype in T2D and these macrophages are an abundant source of cytokines. To date, transcriptome information on the specific cytokine signature of resident and infiltrating macrophages in human T2D islets is still missing. Recent technical advances made single cell RNA sequencing feasible. A study comparing islet cells from T2D individuals and healthy controls found elevated cytokine signaling pathways and MHC II expression in acinar and ductal cells [18]. Two additional single cell RNA sequencing studies focused on endocrine cells and also did not include the rare and very diverse islet immune cells [39, 40]. Despite the tremendous technical advances in single cell RNA sequencing, this technique still has its limitations, as revealed by a recent study by the Kaestner group in which the overlap of differentially expressed genes in the three available single cell RNA sequencing data sets described above was extremely poor [41].

The first causal link between inflammation and in vivo islet function in T2D was established in humans with a double-blinded two-center clinical trial with anakinra, a recombinant human IL-1Ra [4]. In this study, the ratio of insulin to proinsulin in plasma increased with anakinra treatment which is indicative for an improved β-cell function. For obvious reasons, it is not possible in humans to directly investigate the pancreas and islets. Therefore, mouse models are used to shed light on the mechanism of islet failure in T2D (see “Cytokines and chemokines in islets of rodent models of T2D”).

Altogether, numerous studies reporting islet infiltration with proinflammatory-skewed macrophages and presence of elevated cytokines and chemokines in islets from T2D patients strongly support the concept that insulitis governed by activation of the IL-1 system is part of the aetiopathology of T2D in humans. In line with this concept are outcomes of several clinical trials with IL-1 blockage to inhibit inflammation, which resulted in improved insulin secretion and blood glucose control [4, 42]. These clinical intervention studies are the focus of another review by Y. Kataria et al. in this issue of *Seminars in Immunopathology*.

### Cytokines and chemokines in islets of rodent models of T2D

In humans, investigation of islet inflammation is restricted to specimens harvested from organ donors or from surgical pancreas resections of T2D patients and observations therefore remain mostly correlative. Using rodent models of T2D, preventive or interventional studies can be performed and genetic models can be used to target specific genes and molecular pathways followed by direct examination of the pancreata and islets. In most rodent studies, anakinra (antagonizes both IL-1β and IL-1α), specific anti-IL-1β antibodies, or TNF-α blockers were applied in a preventive fashion in diet-induced mouse obesity models or in the GK rat [19, 27, 43–45]. In an initial, preventive study, mice were fed a high-fat diet for 12 weeks and concomitantly treated with anakinra. No effect was observed on β-cell mass, although insulin secretion from isolated islets was improved, β-cell proliferation increased, and β-cell apoptosis reduced in anakinra-treated mice [43]. Treatment of 1-month-old GK rats for 4 weeks with anakinra improved hyperglycemia, insulin sensitivity, prevented immune cell infiltration into islets, and reduced cyto- and chemokine expression in isolated islets, liver, and adipose tissue [27]. An interventive study used a specific anti-IL-1β antibody (XOMA 052) in mice fed a high-fat diet for 10 weeks followed by another 9 weeks with anti-IL-1β therapy. This resulted in improved glucose tolerance and plasma insulin, increased β-cell proliferation, and decreased apoptosis along with increased β-cell mass [44]. In a later study, islets were isolated from mice fed a high-fat diet for 10 weeks followed by 8 weeks of anti-IL-1β treatment. Islets from mice with IL-1 blockage showed increased insulin content and glucose-stimulated insulin secretion [19]. Short-term (2 weeks) anti-IL-1β treatment of db/db mice as well as high-fat-diet-fed mice injected with a single-low dose of the β-cell toxin streptozotocin (a model to mimic diminished β-cell mass as observed in human T2D) also resulted in improved function of isolated islets. In the same study, 14-day treatment with etanercept, a TNF-α blocker, similarly improved islet insulin secretion in the obesity-single-low dose streptozotocin model [19]. Improved β-cell function and reduced inflammation in isolated islets after in vivo treatment with IL-1Ra was also observed in a rodent model of islet amyloidosis [45]. In this model, the amyloidogenic human islet amyloid polypeptide (IAPP) is transgenically expressed in β-cells which leads to plaque formation, activation of the inflammasome, and subsequent maturation and secretion of IL-1β. Genetic ablation of the NLRP3 inflammasome diminishes processing of proIL-1β to mature (active) IL-1β and increases islet area at 1 year of age, implicating IL-1β in the regulation of islet mass [46].

To determine the cellular source of inflammatory factors in islets, clodronate liposomes were used to deplete macrophages from db/db and KKAy mice. This improved glucose tolerance and insulin secretion in vivo and in isolated islets [23]. Similar results were obtained upon macrophage depletion in human IAPP-expressing obese mice [47] and in the Zucker diabetic fatty rat model [25], strongly supporting the current scientific view that infiltrating and resident proinflammatory macrophages are the main source of deleterious islet inflammation in animal models of T2D. Interestingly, recent transcriptome studies and FACS profiling showed that islet-resident macrophages from normal mice are M1-polarized and thus already are in an activated state, a feature which is similar to barrier macrophages of the intestine and the lung. Islet macrophages basally also express high levels of *Tnf*, *Il1b*, and MHC-II when compared to pancreatic stromal macrophages [48] or non-barrier macrophages and it is hypothesized that they are activated by both intra-islet and blood-borne pathogens [48, 49]. Along these lines, the β-cell hormone insulin enhances the proinflammatory M1-polarization state of macrophages [50].

In most rodent studies, IL-1 blockage not only improved islet function and mass but also increased peripheral insulin sensitivity [27, 43, 44]. TNF-α inhibition, which primarily targets insulin-responsive tissues [51], also improved insulin sensitivity. Improved insulin sensitivity decreases insulin demand and therefore reduces the “workload” of β-cells. According to this “β-cell rest” concept, the β-cells may recover and thereby restore their secretory function [52, 53]. Hence, it is not possible to distinguish whether the above described beneficial effects of IL-1 blockade on islet β-cells in vivo are only due to direct inhibition of IL-1 action on β-cells or whether the improvements are also indirect and a consequence of β-cell rest.

Different lines of evidence argue for the importance of direct protective effects of IL-1 blockade on islet β-cells in these rodent models. First, a large number of in vitro studies show direct and deleterious IL1 effects on islet β-cells [30, 54]. A limitation of many of these studies with cultured islets is that pharmacological IL-1β concentrations were used, which exceed both physiological IL-1β concentrations and levels reached in extreme models such as sepsis. Second, whole islets express a functional IL-1 system including the IL-1Receptor (IL-1R1), the agonistic ligands IL-1β and IL-1α, and IL-1Ra [55]. Importantly, mouse β-cells express the highest density of IL-1R1 when compared to other tissues in the mouse and the IL-1R1 is the most abundant cell surface receptor on mouse β-cells [34, 56], suggesting that islet β-cells are a prominent site for IL-1 action. To dissociate indirect peripheral IL-1 effects from direct effects on islets, we targeted the islet local IL-1 system by using a genetic mouse model with β-cell-specific deletion of the protective IL-1Ra [55]. This reduced islet local IL-1Ra expression and release, but not circulating IL-1Ra, and thus increased IL-1 signaling specifically in β-cells. Indeed, these mice show reduced insulin secretion in vivo and in isolated islets, have diminished β-cell proliferation, and lower β-cell area along with an increased number of small islets. Reduced β-cell mass and a disproportionate loss of larger islets is also characteristic for the morphological changes of human islets in T2D [57, 58]. Interestingly, in the β-cell-specific IL-1Ra knockout mice, the deleterious effects on β-cell function and islet size were not due to an overall increase of inflammation in islets, rather, they were due to a direct impact of IL-1β on β-cells, suggesting that increased IL-1β action per se has the potential to impair the morphology and function of mouse β-cells in vivo [55].

## What triggers islet inflammation?

### What triggers islet inflammation in human islets

Naturally, most data concerning triggers of inflammation in human islets stem from in vitro cultures of isolated islets from organ donors. As such, exposure of islets to high glucose levels induced FAS expression [59] and increased production and secretion of IL-1β, while secreted protein levels of other cytokines seemed to be less affected by glucose [8, 10]. These glucose-induced effects could be mediated directly by acting on immune cells or via glucose-induced upregulation of the islet amyloid system, which is a strong activator of islet inflammation (see below). Saturated fatty acids also have been reported to trigger inflammation in human islets. Co-culture of islets from healthy donors with palmitate induced release of the proinflammatory cytokines IL-6 and IL-8 as well as the chemokine CXCL1 [9, 12, 34]. Expression of other cytokines such as IL-1β and TNF-α were also increased by co-culture with palmitate [12]. Oleate and stearate also increased expression levels of cytokines and chemokines [34], although this was not observed in all studies [12]. These free fatty acid-induced effects were blocked by adding IL-1Ra or anti-IL-1β antibodies [34] suggesting that IL-1 signaling is mediating these deleterious effects. Combination of high glucose concentrations and free fatty acids even further stimulated IL-1β expression [34] and the release of chemokines from human islets [9], which may recruit additional macrophages to the islet. Further, IL-1β induces its own expression [10] leading to a vicious cycle and eventually prolonged inflammation. One of the strongest inducers of IL-1β is islet amyloid polypeptide (IAPP, also called amylin), a β-cell-derived hormone that exerts beneficial properties on metabolism, mostly via central induction of satiety. However, under certain conditions, IAPP aggregates and forms toxic plaques (“islet amyloid deposits”). These deposits are found in most individuals with type 2 diabetes [57, 60, 61] and correlate with β-cell apoptosis [62] and reduced β-cell mass, a hallmark of type 2 diabetes [57, 63]. Interestingly, both glucose [64] and free fatty acids [23] promote islet amyloidosis in human islets via upregulation of IAPP expression. IAPP and insulin are co-secreted from β-cells [65, 66] and since insulin secretion is increased in prediabetes to compensate for increased insulin demand [67], IAPP secretion is increased as well. Among other mechanisms, such as insufficiently processed IAPP that is found in failing human islet grafts [68], the increased concentration of IAPP might be a trigger for amyloid deposition. Although in vitro work and studies using transgenic animal models have shed light on many aspects of islet amyloidosis (see “What triggers islet inflammation in rodent models of T2D”), there is currently no therapy available to counteract this contributing factor to loss of β-cell mass in T2D. Further, anti-diabetic drugs that act as insulin secretagogues (like sulfonylurea, GLP-1 agonists, etc.) might partly promote amyloid-induced β-cell apoptosis by increasing β-cell secretion. The specific TLR2 receptor agonist Pam2 induced *IL1b*, *IL6*, and *IL8* expression in human islets and purified human β cells [34]. LPS, a TLR4 receptor agonist, also induced cytokines, although to a lesser extent [34]. TLR receptors are pattern recognition receptors and they are thus not very specific and typically induce an inflammatory response as a reaction to microbial pathogens or lipids, for instance contained in the food. Fatty acids also signal through TLR2 and 4 [69]. Thus, the abovementioned fatty acid-induced increase in IL-1β expression in human islets might at least partly be mediated by TLR signaling. An additional mechanism of proinflammatory cytokine stimulation in human islets is the renin-angiotensin system. Indeed, exposure of human islets to angiotensin 2 induced gene expression of IL-6 and the chemokine MCP-1. These effects were independent of vasoconstriction but IL-1-dependent [26]. Endocannabinoids were also shown to have proinflammatory properties in human tissues. Exposure of human macrophages to the endocannabinoid anandamide induced the expression of NLRP3 inflammasome components and the release of IL-1β and IL-18 [25]. In contrast, exposure of whole human islets to anandamide only slightly induced the expression of proinflammatory factors [25]. This suggests that endocannabinoids act on islets via CB1r signaling on islet macrophages. Finally, co-culture of human islets with the adipocyte-derived leptin reduced IL-1Ra expression and induced IL-1β release [32], suggesting that leptin is also able to off shift the balance of IL-1β to IL-1Ra towards a more proinflammatory state.

### What triggers islet inflammation in rodent models of T2D

Prior to the first reports showing a glucotoxic effect in human islets [8], work in the diabetes-prone *Psammomys obesus* showed that hyperglycemia is associated with islet destruction in vivo and that exposing isolated islets from this gerbil to high glucose induced apoptosis [70]. Similar to findings in human islets, it was subsequently shown in mouse islets that glucose, fatty acids, agonists for toll-like receptor TLR2/6 and 4, angiotensin 2, IAPP, and endocannabinoids induce the expression of chemokines and proinflammatory cytokines [9, 23, 25, 26, 34, 71–73]. The primary role of β-cells is to produce and secrete insulin in response to metabolic needs. Therefore, β-cells have a highly developed endoplasmatic reticulum (ER) where proinsulin is properly folded [74]. However, prolonged high demand, as observed in a prediabetic condition, can lead to ER stress and subsequent inflammation [75]. Palmitate also induces ER stress in mouse and human islets [76]. A recent report shed light on another local activator of islet inflammation, namely the β-cell itself. These data suggest that local resident macrophages sense β-cell activity by reacting to ATP which is co-secreted with insulin by β-cells, and that this leads to macrophage activation and an inflammatory response [77]. One major difference between mouse and human islets in terms of immunological potential is that rodent IAPP does not aggregate to form amyloid deposits [78]. This is due to substitutions in three amino acids located in the region that is detrimental for IAPP aggregation. Therefore, co-cultures with synthetic human IAPP or mice that transgenically express human IAPP are used to decipher the underlying mechanisms. These models have shown that human IAPP acts on both islet endocrine and islet immune cells. In non-immune cells, human IAPP promotes chemokine release [79] which recruits additional macrophages to the islets. In immune cells, IAPP triggers a strong proinflammatory response, including the upregulation of IL-1α and TNF-α and secretion of IL-1β in a NLRP3-dependent manner and via TLR2 signaling. Further, IAPP induces an increased release of various chemokines including CCL2, CCL3, CXCL1, CXCL2, and CXCL10 [47, 79, 80]. Clodronate-mediated depletion of macrophages in human IAPP transgenic mice strongly reduced islet gene expression of the proinflammatory markers *Il1b*, *Nlrp3*, *Ccl2*, and *Tnf*, suggesting that in vivo the main proinflammatory effect of IAPP signals via immune cells [47]. Further, transgenic expression of human IAPP in mouse β-cells skewed islets towards a proinflammatory phenotype and impaired β-cell function [47, 79]. Interestingly, in another study using human IAPP transgenic mice, long-term high-fat diet feeding alone was not able to induce islet inflammation but in combination with the expression of human IAPP, macrophage infiltration, and expression of islet chemokines and proinflammatory cytokines were strongly elevated [81]. Free fatty acids were also shown to directly enhance aggregation of IAPP aggregation in isolated human IAPP transgenic islets [82]. Activation of the FAS receptor [83] and the receptor for advanced glycation end products (RAGE) [84] were also implicated as potential mediators of amyloid-induced toxicity. This suggests that islet amyloid formation is essential to induce islet inflammation, a feature that is underestimated in rodent studies as they do not express amyloidogenic IAPP. Further, addition of IL-1Ra in vivo has been shown to prevent human IAPP-mediated upregulation of islet gene expression of IL-1α, Il-1β, TNF-α, and CCL2 [45]. IL-1Ra also had beneficial effects in other mouse models of T2D, such as the prevention of high-fat diet-induced hyperglycemia and glucose intolerance in wild-type [43], db/db and KKAy mice [23], and the GK rat [27]. Together with the anti-diabetic action of macrophage depletion by clodronate in mice [23, 47] and the Zucker rat [25], these data show that IL-1 receptor signaling is an essential part of the metabolic syndrome.

## Adaptive and physiological function of islet immune cells and cytokines

Inflammation primarily serves to restore homeostasis upon an insult on the integrity of an organism. Invasion of an organism with foreign pathogens or an injury are classical disruptors of homeostasis. As long as inflammation resolves and is limited in time, it is adaptive and restorative and protects the organism. Only when inflammation is chronic or excessive it becomes pathological and promotes various associated diseases. Indeed, the state of chronic low-grade inflammation induced by metabolic stress due to persistent nutrient overload is causally linked to metabolic diseases such as obesity, atherosclerosis, and T2D [3, 80, 85–89]. The vast majority of published data related to the interaction of the immune system with metabolism focused on these deleterious aspects of metabolic inflammation while the homeostatic and restorative functions of innate immunity became only recently a focus of research [3]. Indeed, macrophages were shown to have a trophic role in the development of the pancreas and fetal endocrine cells, both in humans and in mice [90–92]. Op/op mice, which are CSF-1-deficient and lack macrophages, have a reduced β-cell mass and impaired islet morphogenesis [90]. Macrophages also play an important role in the adaptation to injury in the pancreatic duct ligation model [93]. Ablation of macrophages by clodronate liposomes in this regeneration model reduced the infiltration of islets with macrophages and completely prevented β-cell replication following injury. Profiling of these macrophages indicated that anti-inflammatory M2 macrophages exert these functions. Further, mesenchymal stem cell transplantation into diabetic mice induced recruitment of M2-polarized macrophages, which in turn promoted β-cell regeneration [94]. In a mouse model of vascular endothelial growth factor-A-induced loss of β-cells, recruited macrophages are also necessary for β-cell regeneration [95]. Further, in obese mice, islet macrophages promote adaptive β-cell proliferation via the platelet-derived growth factor receptor. However, at the same time, these macrophages inhibit insulin secretion in a contact-dependent manner [29]. These examples illustrate that resident and infiltrating islet macrophages can exert homeostatic and regenerative functions, probably via alteration of macrophage polarization. Hence, it is context dependent if innate immune cells adopt beneficial or pathological features.

Besides macrophages, the recently characterized rare group 2 innate lymphoid immune cells (IlC2s), which are induced by IL-33, promote β-cell function both in normal mice and upon induction of diabetes with the β-cell toxin streptozotocin [96]. In contrast, IL-33 knockout mice have impaired insulin secretion. Interestingly, the IL-33 receptor (IL1RL1) and IL-33 are among the 100 most highly upregulated genes in a transcriptome study of human islets with T2D (IL1RL1 rank 6 and IL33 rank 26) pointing to a need for activation of restorative pathways in human T2D [13].

IL-22, which belongs to the IL-10 superfamily, is another cytokine derived from ILCs, T helper cell subsets, and natural killer T cells that also have beneficial effects on insulin secretion. Both pharmacological and endogenous IL-22 partially prevented ER and oxidative stress induced by glucolipotoxicity in pancreatic β-cells and thereby improved insulin secretion [37].

Context-dependent homeostatic, beneficial roles on β-cell function and islet morphology have not only been described for resident immune cells but also for cytokines and chemokines that are elevated in the circulation of patients with T2D. Indeed, increased IL-6 levels in response to obesity or physical exercise promotes the secretion of the incretin hormone GLP-1 by intestinal L-cells and pancreatic α-cells leading to increased insulin secretion [97]. Thereby, IL-6 contributes to the adaptation of β-cell function to the increased insulin demand in physiology and obesity [97]. The toxicity of IL-1β for β-cells was a major focus of research in the past decade [30, 54, 98], although a dual concentration-dependent role on insulin secretion was observed in isolated islets already three decades ago [98]. Indeed, IL-1β is an insulin secretagogue in the context of food intake and adaptation to the increased insulin demand upon high-fat diet feeding [50, 99]. During food intake, circulating IL-1β produced by myeloid cells increases and contributes to meal-induced insulin secretion in a fasting-refeeding mouse model. Further, IL-1β-induced insulin secretion was diminished in diabetic mice transplanted with islets lacking the IL-1R1 relative to mice transplanted with normal islets [50]. Mice lacking the IL-1R1 had an impaired adaptive increase of plasma insulin upon short-term high-fat diet feeding [99]. Interestingly, in mice, insulin reinforces a proinflammatory state in macrophages by stimulating IL-1β production via glucose uptake and activation of the NLRP3 inflammasome. It is not known to date whether postprandial IL-1β similarly promotes insulin secretion in humans. However, also in cultured human islets [50, 99] and human ENDOC cells [50] IL-1β triggers insulin secretion.

The above described examples illustrate how innate immune cells and their products modulate endocrine cells to shape them for stress and to regulate their development and regeneration. These homeostatic functions have to be taken into consideration when developing immunomodulatory therapies.

## What causes islet inflammation to switch from a physiologic to a pathological role?

In the scientific community, the widespread view predominates that products of the innate immune cells mainly serve to regulate the immune system. Indeed, the innate immune system is a first-line defense against foreign pathogens and in higher organisms also an activator of the adaptive immune system. However, cytokines and chemokines have signaling properties in many other non-immune cell types and regulate their function, development, and intra-organ communication in a physiological context. A prime example is the prototypical cytokine IL-1β, which is mainly known for its role in the amplification of immune responses in mammals. However, already in evolutionarily more ancient organisms like the fish, which possess no adaptive immunity, a single IL-1β homolog exists [100], which acts as a signaling molecule in the neuro-endocrine system [101]. It activates the hypothalamic-pituitary-interrenal axis and promotes cortisol release in fish [102]. This ancient effector function of IL-1β on neuro-endocrine cells is preserved in mammals where IL-1β similarly regulates the hypothalamic-pituitary-adrenal axis [103, 104]. Further, in the frog *Xenopus laevis*, IL-1β appears to play a role in the development of neuronal circuits [105], a function not directly related to immunity. Neuro-endocrine and pancreatic β-cells express similar neuronal transcription programs and share many functional and developmental properties [106]. Furthermore, the IL-1R1 is prominently expressed in many non-immune cells including endothelial cells, the pituitary, and the pancreatic β-cells [107]. Hence, IL-1β is a very ancient endocrine regulator, which adopted its amplifying role in the mammalian immune system only later in evolution. It is thus not surprising that β-cells with their prominent IL-1R1 expression are physiological targets of IL-1β signaling.

How then can the physiological function of IL-1β in postprandial insulin secretion be reconciled with its pathological role in β-cell dysfunction and demise? There are several possible explanations for this dual effect of IL-1β on β-cells. One explanation is the consequence of acute and short-term versus chronic stimulation of insulin secretion. Chronic stimulation of insulin release by IL-1β may eventually exhaust the β-cells. Blocking IL-1β therapeutically may allow β-cells to rest and recover [52, 53], as discussed above. Second, chronic stimulation may lead to non-responsiveness or resistance to IL-1β. Indeed, in contrast to healthy islets, islets from T2D donors do not secrete insulin in response to low-dose IL-1β [99]. Third, there may be insufficient IL-1β counter regulation in islets. Islet β-cells endogenously express the protective factor IL-1Ra [32, 33, 55], which is induced by IL-1β itself to form a negative feedback loop. Mice with constitutive β-cell-specific IL-1Ra knockout have impaired insulin secretion in response to a high glucose bolus [55]. Further, human T2D islets or cultured islets exposed to glucotoxicity have diminished IL-1Ra expression and this may lead to chronic exposure of β-cells to IL-1β [10, 32]. Fourth, long-term exposure and higher levels of IL-1β may eventually induce changes in transcriptional programs, which regulate β-cell identity, proliferation, and apoptosis. Since the rate of β-cell turnover is very low, it may take a rather long time for negative effects on β-cell mass to manifest. In agreement with this, T2D in humans typically develops over years or even decades. Constitutive deletion of the protective IL-1Ra in mouse β-cells indeed mainly reduces proliferation genes and diminished glucose-induced insulin secretion. This impairment was partly rescued by overexpression of the transcription factor E2F1. Of note, in β-cells, this master cell cycle regulator E2F1 not only targets proliferation genes but directly regulates the promoter of the potassium channel subunit Kir6.2, which is crucial for insulin secretion [55]. This suggests that inhibition of proliferation genes in β-cells not only affects islet size but also impairs β-cell function.

## Conclusion

**Fig. 1:**
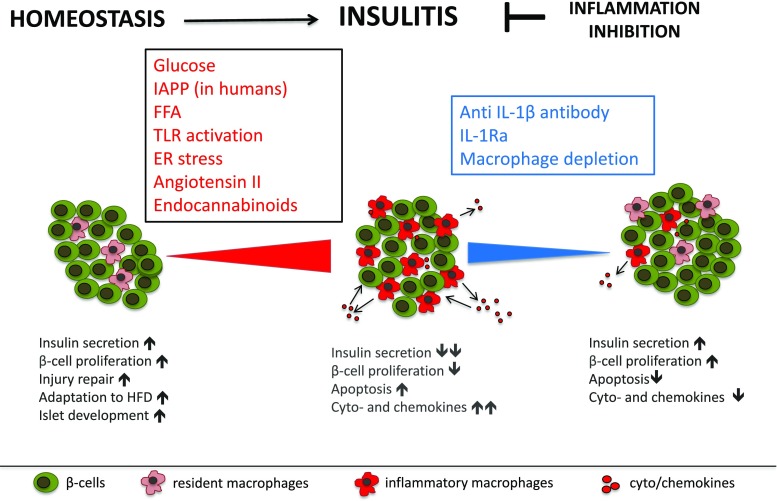
In physiology, resident macrophages and cytokines have a homeostatic role in the development and function of islet β-cells. T2D is associated with chronic, low-grade inflammation in pancreatic islets. Insulitis is characterized by an elevated number of proinflammatory macrophages and increased levels of cytokines and chemokines and contributes to impaired islet function. Depletion of macrophages and inhibition of the IL-1 system in islets reduces macrophage infiltration and proinflammatory cyto- and chemokine expression and improves insulin secretion

It is established nowadays that insulitis characterized by elevated numbers and activity of mononuclear cells and of increased proinflammatory factors is part of the aetiopathology of T2D (Fig. 1). Further, based on studies with IL-1 antagonism in humans and rodent models, we know that the IL-1 system drives islet inflammation. However, there are still many open mechanistic questions that need to be addressed in future research. It is still unclear what the origin of the increased islet immune cells is, whether they are recruited to the islets, or whether they mainly stem from resident immune cells. Further, it has not yet been elucidated how exactly the endocrine cells signal to the immune cells and change their activity and whether they contribute to the recruitment and activation of immune cells. Also, there is limited knowledge on the physiological signaling function of cytokines and chemokines and what the cellular sources are within the islet. Since anti-inflammatory treatments may be clinically implemented in the near future, it will be important to understand the role of inflammation in both islet biology and pathology.
